# Metagenomic analysis of nitrogen‐cycling genes in upper Mississippi river sediment with mussel assemblages

**DOI:** 10.1002/mbo3.739

**Published:** 2018-10-01

**Authors:** Ellen M. Black, Michael S. Chimenti, Craig L. Just

**Affiliations:** ^1^ Department of Civil and Environmental Engineering University of Iowa Iowa City Iowa; ^2^ Iowa Institute of Human Genetics Carver College of Medicine University of Iowa Iowa City Iowa

**Keywords:** freshwater mussels, metagenomics, N‐cycle, nitrification, *Nitrospira*, sediment

## Abstract

We investigated the impact of native freshwater mussel assemblages (order Unionoida) on the abundance and composition of nitrogen‐cycling genes in sediment of an upper Mississippi river habitat. We hypothesized that the genomic potential for ammonia and nitrite oxidation would be greater in the sediment with mussel assemblages, presumably due to mussel biodeposition products, namely ammonia and organic carbon. Regardless of the presence of mussels, upper Mississippi river sediment microbial communities had the largest genomic potential for nitrogen fixation followed by urea catabolism, nitrate metabolism, and nitrate assimilation, as evidenced by analysis of nitrogen cycling pathway abundances. However, genes encoding nitrate and nitrite redox reactions, *narGHI* and *nxrAB*, were the most abundant functional genes of the nitrogen cycling gene families. Using linear discriminant analysis (LDA), we found nitrification genes were the most important biomarkers for nitrogen cycling genomic potential when mussels were present, and this presented an opposing effect on the abundance of genes encoding nitric oxide reduction. The genes involved in nitrification that increased the most were *amoA* associated with comammox *Nitrospira* and *nxr* homologs associated with *Nitrospira*. On the other hand, the most distinctive biomarkers of microbial communities without mussels were *norB* and *nrfA*, as part of denitrification and dissimilatory nitrate reduction to ammonium pathways, respectively. Ultimately, this research demonstrates the impact of native mollusks on microbial nitrogen cycling in an aquatic agroecosystem.

## 1 INTRODUCTION

The estimated, healthy “planetary boundary” for land‐applied nitrogen (N) is 35 teragrams (Tg)‐N/year (Galloway et al., 2004; Kroeze, Mosier, & Bouwman, 1999; Rockstrom et al., 2009). Human activity has pushed the planet well beyond this boundary, to 150 Tg‐N/year (Ollivier et al., 2011), resulting in excessive aquatic eutrophication and harmful algal blooms (Glibert, Maranger, Sobota, & Bouwman, 2014). Excess land‐applied urea and ammonia (NH_3_) are biologically oxidized to nitrate (${\text{NO}}_{3}^{-}$), which has a high runoff potential (Sebilo, Mayer, Nicolardot, Pinay, & Mariotti, 2013), causing negative ecosystem impacts, degraded water quality, and biogeochemical cycling imbalances (NRC, 2015). The well‐studied Upper Mississippi River (UMR) basin (Burkart & James, 1999; Lerch, Kitchen, Baffaut, & Vories, 2015), that contributes 50,000 metric tons of N to the Gulf of Mexico annually (Donner & Kucharik, 2008), is a global “epicenter” of excessive N‐transfer from land to water. In the UMR, microbially driven N‐biogeochemistry (Kreiling, Richardson, Cavanaugh, & Bartsch, 2011) is symbiotically linked to freshwater mussel N‐cycling. Billions of native freshwater mussels live in assemblages in the UMR, filter billions of gallons of water, and remove tons of biomass from overlying water daily (Newton, Zigler, Rogala, Gray, & Davis, 2011). Previous studies have shown that mussels increase the concentration of N in sediment porewater via bioactivity including burrowing (bioturbation) and excretion of feces and pseudofeces (biodeposition) (Bril, Durst, Hurley, Just, & Newton, 2014; Bril, Langenfeld, Just, Spak, & Newton, 2017). Intermittent sediment aeration and elevated nutrient concentrations create a niche ripe for removal of N at the interface of oxic and anoxic conditions, via nitrification, denitrification, and anaerobic ammonium (${\text{NH}}_{4}^{+}$) oxidizing (anammox) processes, making mussels and microbial activity a functional biological unit for N‐cycling in aquatic systems.

The main functions of N‐cycling microorganisms in aquatic sediments include N‐fixation by benthic organisms, and elemental transformations such as nitrification and denitrification, which oxidizes and reduces inorganic N, respectively (Barrios, 2007). Biological N‐fixation by benthic prokaryotes commonly produces 0.4–1.6 g‐N m^−2^ year^−1^ and reaches 76 g‐N m^−2^ year^−1^ in dense microbial mats (Howarth, Marino, & Cole, 1988). Microorganisms fix N with the nitrogenase (Nif) enzyme complex and are responsible for catalyzing half of the bioavailable N on Earth (Boyd & Peters, 2013). Bioavailable N (ammonia) can be assimilated into biomass for growth or used as an energy source in nitrifying organisms. The NH_3_ monooxygenase (Amo) enzyme catalyzes the oxidation of NH_3_ into hydroxylamine (NH_2_OH), which may be oxidized to nitrite (${\text{NO}}_{2}^{-}$) using hydroxylamine oxidoreductase (Hao), and complete nitrification occurs when ${\text{NO}}_{2}^{-}$ is oxidized to ${\text{NO}}_{3}^{-}$ in organisms containing a nitrite oxidoreductase enzyme (Nxr). Numerous microorganisms are capable of partial nitrification, the oxidation of NH_3_ to ${\text{NO}}_{2}^{-}$ or ${\text{NO}}_{2}^{-}$ to ${\text{NO}}_{3}^{-}$, while only the *Nitrospira* lineage II contains the genetic potential to completely oxidize NH_3_ to ${\text{NO}}_{3}^{-}$ (Daims et al., 2015; van Kessel et al., 2015). Another metabolic pathway for NH_3_ oxidation is present in anammox bacteria (Planctomycetes phylum) which oxidize ${\text{NH}}_{4}^{+}$, reduce ${\text{NO}}_{2}^{-}$, and produce a hydrazine intermediate with the hydrazine synthase enzyme (Hzs) to ultimately produce N_2_ gas (Oshiki, Satoh, & Okabe, 2016). Competition for N resources arises from the metabolic pathways of dissimilatory ${\text{NO}}_{3}^{-}$ reduction to NH_3_ (DNRA), stepwise ${\text{NO}}_{3}^{-}$ reduction to NOx (${\text{NO}}_{2}^{-}$, nitric oxide, nitrous oxide), or complete denitrification to N_2_.

N‐cycling ecosystem services are impacted in agroecosystems due to the increased availability of reactive N (Hayatsu, Tago, & Saito, 2008). For example, NH_3_ oxidizing pathways are enhanced by greater NH_3_ concentrations, and the subsequently oxidized‐N also enhances nitrate reduction pathways. However, more research is needed to accurately quantify services of biogeochemical cycling in agroecosystems (Zhang, Ricketts, Kremen, Carney, & Swinton, 2007), especially in aquatic systems where macrobiota significantly enhance the transfer of N from overlying water to sediment.

In a previous study, we showed that sediment underlying a native freshwater mussel assemblage harbored microbial communities with lower species richness and evenness as compared to mussel‐free sediment (Black, Chimenti, & Just, 2017). Additionally, mussels had a distinct and significant effect on the vertical distribution of multiple N‐cycling microorganisms, including ${\text{NO}}_{2}^{-}$ oxidizing bacteria (NOB) in the genus *Nitrospira,* aerobic NH_3_ oxidizing bacteria (AOB) in family Nitrosomonadaceae, and anammox bacteria from candidate genus *Brocadia*. Anammox taxons were increased most drastically at 3 cm depth below the water–sediment interface, a depth which is relevant to mussel burrowing, and suggested the presence of an oxic‐anoxic interface niche for N‐cycling microorganisms 3 cm below the water–sediment interface. The abundance of anammox bacteria was the most similar between the shallow (3 cm) mussel sediment and deeper (5 cm) no‐mussel sediment. Therefore, these two sample groups were chosen for follow‐up metagenomic sequencing to assess how mussel presence impacted N‐cycling gene abundances in N‐cycling communities of an anammox niche.

This study aimed to determine if mussels increased the abundance of N‐cycling genes, especially genes responsible for NH_3_ oxidation, ${\text{NO}}_{2}^{-}$ oxidation, and would clarify previous findings of increased AOB and NOB taxons with mussels. Our hypothesis was that N‐cycling microbial communities of the previously determined oxic–anoxic (anammox) interface niche will contain greater metabolic potentials for urea degradation, NH_3_ oxidation, and ${\text{NO}}_{2}^{-}$ oxidation reactions in the presence of mussels. These results would indicate which N metabolic pathways are most impacted by mussel assemblages in the UMR.

## 2 MATERIALS AND METHODS

### 2.1 Sediment collection and DNA isolation

Sediment cores were removed from the well‐established mussel assemblage in the buffalo habitat (41.452804, −90.763299) in the Upper Mississippi River and a slightly upstream site (41.451540, −90.753275) without mussels using a 2‐in diameter, post‐driver with a polypropylene liner (Multi‐State Sediment Sampler, Art's Manufacturing and Supply, Inc.; American Falls, ID, USA). Sediment samples were removed from collected cores using an ethanol flame‐sterilized 3/8‐in diameter drill bit at sediment depths of 3 and 5 cm. Sediment (0.25 g) was removed in quadruplicate (*n* = 4, 3 cm depth with mussels; *n* = 4, 5 cm depth without mussels) and stored in sterile bead‐beating tubes overnight at −20°C. Genomic DNA was isolated (PowerSoil DNA Isolation Kit; MoBio Laboratories, Inc., Carlsbad, CA, USA), assessed for total DNA quality and quantity (NanoDrop 1000; Thermo Fisher Scientific, Waltham, MA), and stored at −20°C prior to sequencing. These samples correspond to representative sequences without mussels and with mussels (Supporting Information Table S1) from a previous 16S rRNA amplicon study of N‐cycling taxonomic profiling (Black et al., 2017).

### 2.2 Metagenomic shotgun sequencing

For each sample, 120 ng of genomic DNA in 60 μl of 10 mM Tris‐HCl, pH 8.0 buffer, was placed into 1.5 ml RNase‐/DNase‐free, low‐binding microcentrifuge tubes. Library creation steps were performed by the University of Iowa Institute for Human Genetics, Genomics Division and included DNA shearing using the Covaris Adaptive Focused Acoustics^™^ process (Covaris E220 Focused‐ultrasonicator; Covaris, Inc., Woburn, MA), and DNA fragment purification and end polishing (KAPA Hyper prep kits; Kapa Biosystems, Inc., Wilmington, MA) prior to ligation to indexed adaptors. The library size distribution was validated using the Agilent 2100 Bioanalyzer Instrument (Agilent Technologies, Santa Clara, CA) and quantified using the q‐PCR KAPA library amplification module following manufacturer instructions (Kapa Biosystems, Inc.). The indexed libraries were normalized, pooled, and clustered on a flow cell using the cBOT Cluster Generation System (Illumina, Inc., San Diego, CA) and sequenced on the Illumina HiSeq 4000 System (Illumina, Inc.) in high output mode (1 lane, 2 × 150 bp). Metagenomic reads and sequence statistics are accessible at the MG‐RAST server, European Nucleotide Archive, and the NCBI Sequence Read Archive (Supporting Information Table S1).

### 2.3 Bioinformatics pipeline

Using the HUMAnN2 (Abubucker et al., 2012) standard workflow, paired‐end reads were imported into MetaPhlAn2 (Segata et al., 2012) and mapped against functionally annotated genomes from the ChocoPhlAn pangenome database (NCBI RefSeq Release 80) using Bowtie2 algorithm with default settings (Langmead & Salzberg, 2012). Unmapped reads were subjected to rapid translated search using the Diamond (Buchfink, Xie, & Huson, 2015) algorithm against the universal protein reference database for 90% similarity (UniRef90 (Li & Godzik, 2006)) within HUMAnN2 using default values (evalue threshold = 1.0, prescreen threshold = 0.01, and identity threshold = 50.0%). Hits to protein families and organism‐specific gene hits were compared to the 2016 *MetaCyc* pathway collection (Caspi et al., 2016) using HUMAnN2 core algorithms. The output of this pipeline included tables of gene family and pathway abundances in units of reads per kilobase (RPK) and pathway coverages. Gene and pathway abundance tables were normalized for sample sequencing depth in copies per million (CPM), labeled with “mussel” and “no‐mussel” metadata categories, and stratified by lowest common ancestor (LCA) classification using the scripts “humann2_renorm_table” and “humann2_infer_taxonomy”, as provided with the HUMAnN2 package. The CPM‐normalized gene families were regrouped into Gene Ontology (GO) and KEGG Orthology terms via the “humann2_regroup_table” script and mapping files “uniref90_go” and “uniref90_ko”. Of the original protein clusters, 68.6% and 33.0% were successfully regrouped into GO and KO features for further processing, respectively.

### 2.4 LDA effect size and phylogenetic tree construction

N‐cycling gene families were placed into parent categories of N‐cycle function defined within the KEGG “Nitrogen metabolism” module (i.e. nitrification, denitrification, anammox, N‐fixation, assimilation), and further specified into functional sub‐categories (i.e. ammonia oxidation, nitrite oxidation.), and gene families, as defined by KEGG ontologies (i.e. *amoA, nxrA*; Supporting Information Table S2). Relative abundances (CPM) were assessed for linear discriminant analysis (LDA) effect size (LEfSe), a method to determine the consistent metagenomic features responsible for differences between microbial communities (Segata et al., 2011). All samples were labeled by class (*n* = 4 with mussels, *n* = 4 without mussels), and features were compared for differential distribution using the non‐parametric factorial Kruskal–Wallis rank‐sum test (alpha = 0.05). Features deemed differentially abundant were compared for effect size using the pairwise Wilcoxon rank‐sum test (alpha = 0.05), and input into a LDA model which ranked features according to effect size, with a LDA score of ±2.0 chosen as a cutoff for inclusion as a significant feature. The LEfSe program ranked genes by effect size, with the highest ranking given to those with biological consistency, meaning differential abundance scores held true for higher order categories of gene and pathway abundances. LEfSe biomarker results were graphically displayed with the “Plot Cladogram” command.

All N‐cycle genes identified as differentially abundant were labeled with species of origin from the protein cluster's mapping to NCBI taxonomy ID. The comammox genome from *Candidatus* Nitrospira inopinata was not included in the ChocoPhlAn pangenome at the time of this study. As a result, we used multiple sequence alignments of protein sequences to determine if differentially abundant nitrification functional genes originated from the comammox *Nitrospira* lineage (Supporting Information Figure S1). Multiple sequence alignments of AmoA proteins were performed in MEGA7.0.20 (Kumar, Stecher, & Tamura, 2016) using reference sequences from IMG (Supporting Information Table S3), using the MUSCLE algorithm (Edgar, 2004) with default options (Gap penalties: open = −2.9, gap extend = 0, hydrophobicity multiplier = 1.2), the neighbor joining method of clustering (8 iterations, *γ* = 24) (Edgar, 2004), and trimmed for quality in Jalview (Waterhouse, Procter, Martin, Clamp, & Barton, 2009). Phylogeny was reconstructed using 100 bootstrap replications (Felsenstein, 1985) of maximum‐likelihood method based on the Poisson model for amino acid substitutions, assuming gamma distributed evolution rates with five discrete categories, and 80% site coverage cutoff for partial deletions. Trees were constructed with the Subtree‐Pruning‐Regrafting (SPR) maximum likelihood heuristic method, and the initial tree was inferred by the Neighbor‐Join and BioNJ algorithms (Kumar et al., 2016).

## 3 RESULTS

### 3.1 N‐cycling gene abundances

**Figure 1:**
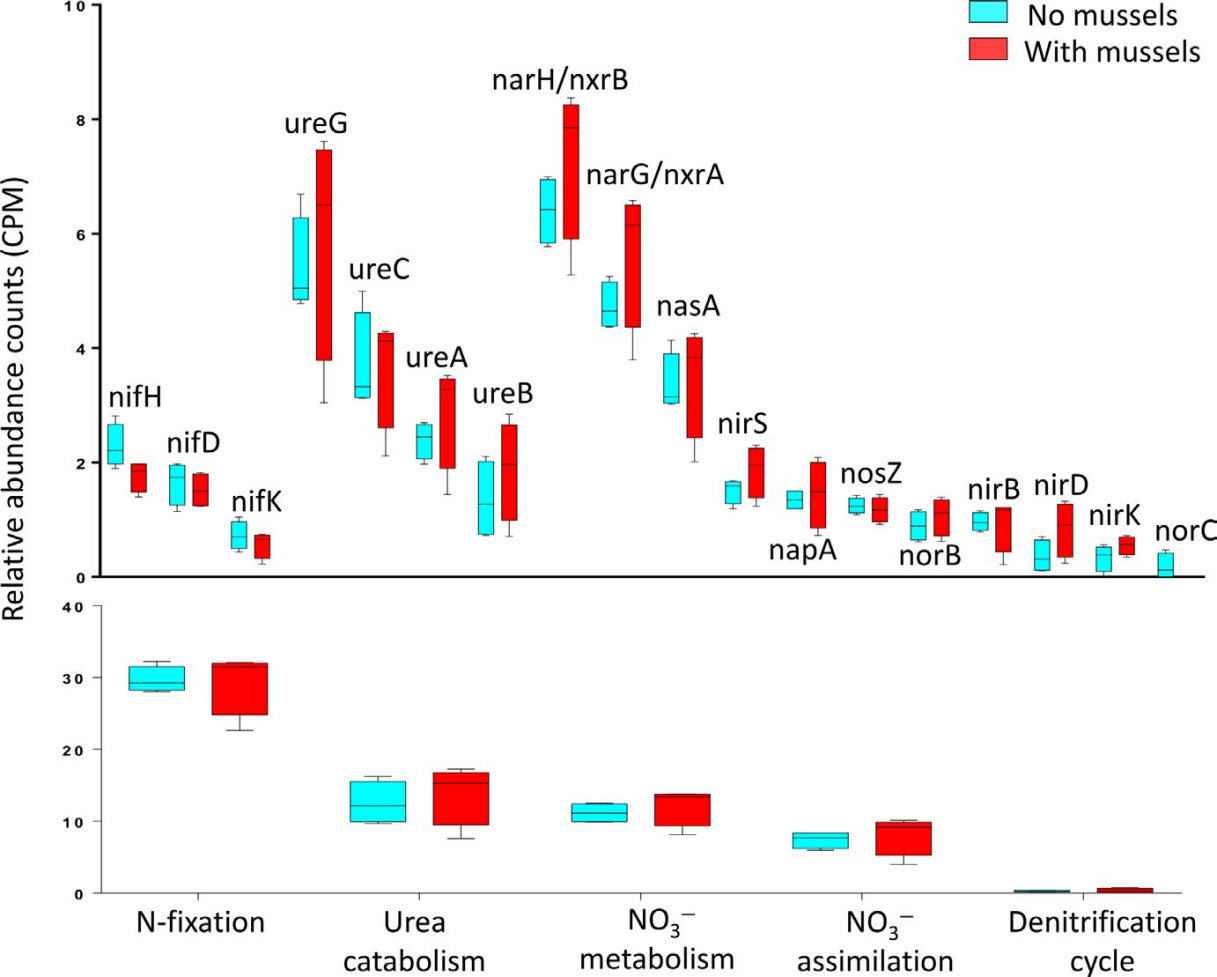
Relative abundance counts of the most abundant N‐cycling pathways (bottom) grouped according to the Gene Ontology database, and the corresponding orthologous groups (above) according to KOs within each pathway

The goal of this research was to determine which N‐cycling processes dominated microbial communities in UMR sediment, and which genes were most characteristic of sediment with and without mussel assemblages. Nitrogen compound metabolic processes (GO:0006807) represented an average relative abundance of 66.0 CPM (±3.1 CPM) in microbial communities with mussels and 63.3 CPM (±3.2 CPM) without mussels and was more abundant than other biological processes such as “aerobic respiration” and “one‐carbon metabolic processes”. According to GO annotations (Figure 1), microbial communities had the greatest potential for N‐fixation even though *nifHDK* was not the most abundant N‐cycling gene family considered in the study. This may be explained by the fact that the GO parent category includes all genes involved in N‐fixation, including non‐biomarker genes encoding N regulation proteins (Ntr), and not solely *nifHDK*. Urea catabolism and ${\text{NO}}_{3}^{-}$ metabolic processes were similarly abundant and were composed of gene families with the largest standard deviations. Gene families specific to the denitrification pathway (*nirK*,* nirS*,* norBC*, and *nosZ*) had smaller standard deviations than other N‐cycling gene families, both within biological replicates and between mussel and no‐mussel treatments. Nitrification biomarkers were moderately abundant, with NH_3_ oxidation representing a majority of the genetic potential. The average abundance of *amoCAB* with mussels was 1.6 (±0.6 CPM), the no‐mussel treatment had an average count of 1.4 (±0.3 CPM), and both treatments had an average abundance less than 1 CPM for *hao*. Both the mussel and no‐mussel metagenomes had non‐detectable abundances of anammox biomarkers, *hzs* and *hdh*. The ${\text{NO}}_{2}^{-}$/${\text{NO}}_{3}^{-}$ transforming gene families (*narGH/nxrAB*) represented the largest N‐cycling gene family but were also quite variable in the treatment with mussels. Ultimately, these microbial communities show large genomic potentials to transform ${\text{NO}}_{2}^{-}$/${\text{NO}}_{3}^{-}$ rather than removing N through denitrification or anammox processes.

### 3.2 LDA effect size

**Figure 2:**
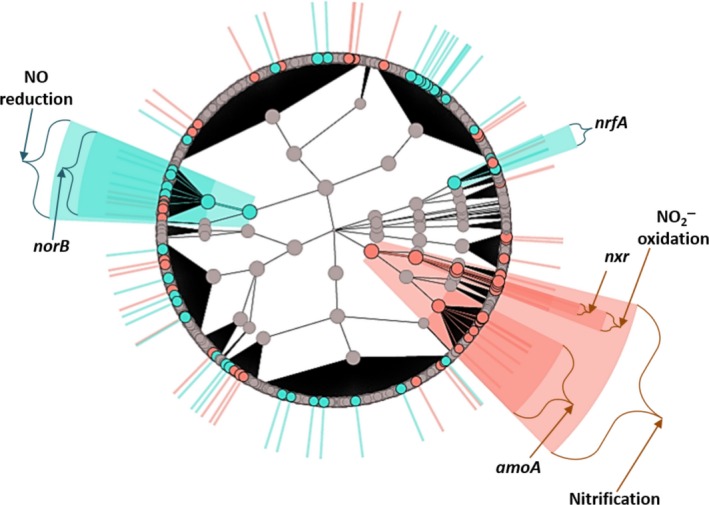
Cladogram of N‐cycling functional genes present in the metagenomic samples, with the outermost circles representing specific UniRef90 protein clusters. Genes were place in functional categories based on KO groups (i.e. *nxr*), enzymatic reactions (i.e. nitrite oxidation), and N‐cycling pathways (i.e. nitrification) as described in the methods. Gene families and functional categories are labeled with colored circles if they were differentially abundant in the treatment with mussels (orange bars) and without mussel (green bars) and are shown with radial extensions beyond the cladogram. Circle sizes represent relative counts (CPM) in each category. Circles near the center represent N‐cycling pathways (defined in Supporting Information Table S2), and categories become more specific as circles are farther from the cladogram center. Genes encoding Nxr and AmoA were the most differentially abundant features with mussels and corresponded to a differentially abundant nitrification pathway. No‐mussel samples were distinguished by increased NO reduction genes and had increased abundances in *norB* and *nrfA* orthologs

**Figure 3:**
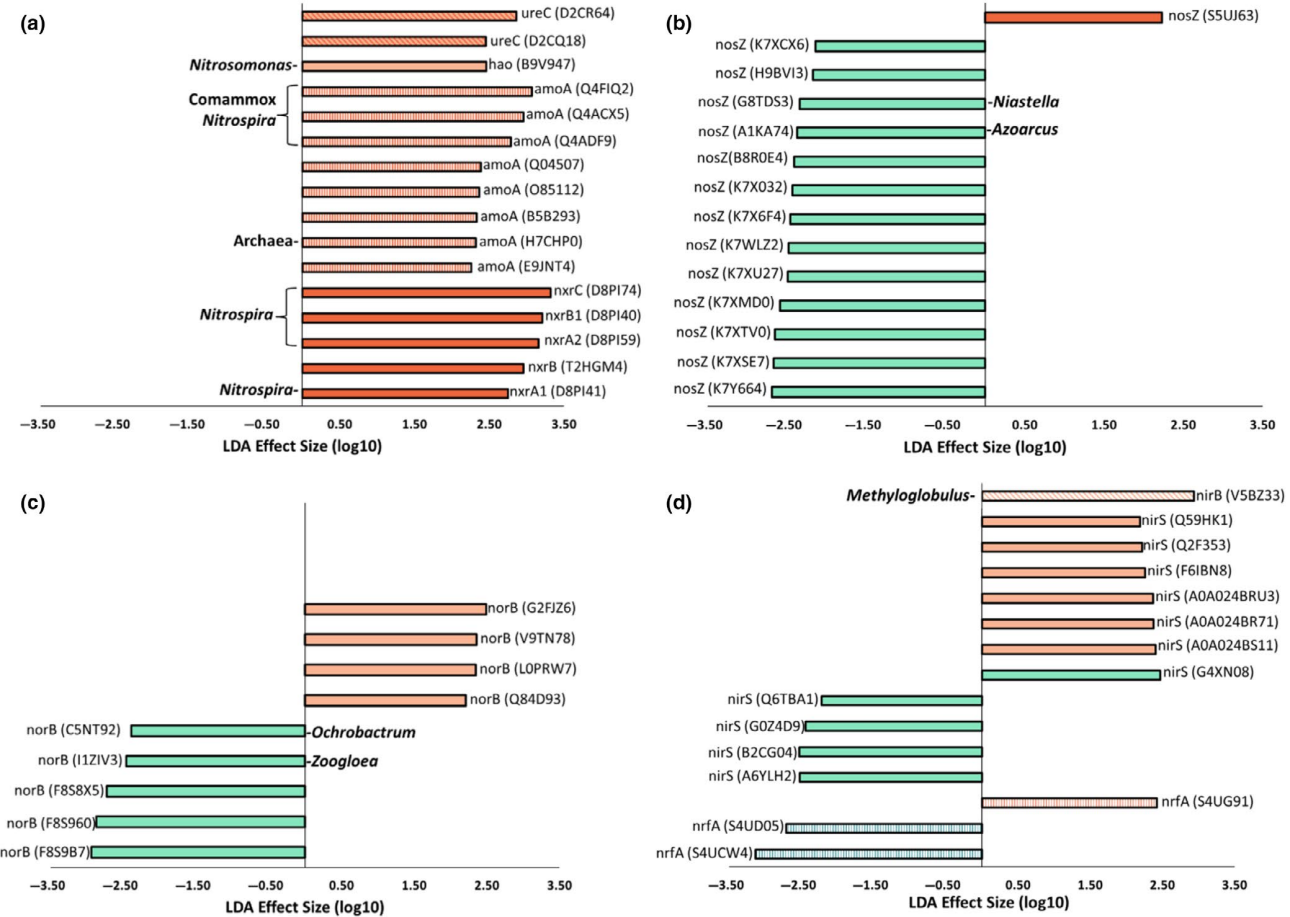
Linear discriminant analysis score for N‐cycle gene families. Each bar represents the effect size for a specific gene cluster, with negative linear discriminant analysis (LDA) scores representing no‐mussel samples, and positive scores corresponding with mussel samples. Genes with an LDA score <2 are not depicted. Each bar represents a gene cluster which is responsible for the distinctness of N‐cycling gene families in mussel treatments. Genes are labeled with taxonomic origin according to mapping of LDA to respective UniRef90 IDs (See [Link], [Link] for comammox *Nitrospira*). Taxonomic origin was not included for genes with no taxonomy designated at the genus level. (a) Nitrification and urea degradation gene clusters with significant LDA scores included genes encoding urease, hydroxylamine oxidoreductase, ammonia monooxygenase, and nitrite oxidoreductase. Abundances of *amoA* and *nxr* genes were most responsible for the 4.3 effect size of the nitrification pathway with mussels. (b) Genes encoding nitrous oxide reduction experienced effect sizes up to 2.7 without mussels. (c) Genes encoding nitric oxide reductases were shown to be a biomarker of no‐mussel metagenomes and had effect sizes up to 2.9. (d) Dissimilatory nitrite reductase genes in the DNRA pathway (*nirB*,* nrfA*) had larger effect sizes than the NO‐forming protein clusters (NirS) belonging to the denitrification pathway

Linear discriminant analysis biomarker discovery was used to identify N‐cycling genes and pathways that were differentially abundant between the mussel treatments. Nitrification functional genes were the most influential biomarker for the mussel metagenomes (Table 1), with ${\text{NO}}_{2}^{-}$ oxidization and *amoA* gene families responsible for most of the increased abundance (Figure 2). For ${\text{NO}}_{2}^{-}$ oxidation, the large increases in *nxrB* and *nxrC* gene abundances were attributed to protein clusters derived from the genome of *Nitrospira* (Figure 3a, Supporting Information Table S3) as were increases in *nxrA2* and *nxrA1*. Of the increased *amoA* genes with mussels, the most differentially abundant *amoA* were similar to protein clusters aligning with comammox *amoA,* as shown through phylogenetic analysis (Supporting Information Figure S1, Supporting Information Table S4). 2 *ureC* genes and 1 *Nitrosomonas hao* gene were increased in abundance with mussels (Figure 3a, Supporting Information Table S3). Although nitrification was the strongest biomarker for microbial communities with mussels, some genes in the denitrification pathway were more abundant with mussels (Figure 3b,c). These included 1 *nosZ* gene and 4 *norB* gene families.

**Table 1:** Biomarker N‐cycling pathways, functional role, and gene families with mussels

	N‐cycling function or functional gene	Linear discriminant analysis effect size	*p* value
Level 1: N‐cycle pathway	Nitrification	4.38	0.021
Level 2: N‐cycle function	Nitrite oxidation	3.98	0.021
Level 3: functional genes	*nxrB*	3.74	0.043
	*nxrC*	3.34	0.018
	*amoA*	3.73	0.021

*Note*

Genes *amoA*,* nxrB,* and *nxrC* were statistically greater with mussels, and the higher order classifications of nitrite oxidation and nitrification were also statistically significant.

N‐cycling genes that increased with mussels originated from taxons known for elemental cycling, such sulfur and methane transformation. For example, differentially increased protein clusters used in dissimilatory nitrate reduction, NarG and NarH (Supporting Information Table S5), were associated with the methane oxidizing *Methylobacter* and methanotrophic *Methylosarcina*, respectively. In another example, one dissimilatory nitrite reducing cluster (NirB; Figure 3d, Supporting Information Table S6) originated from the methanotrophic genus, *Methyloglobulus*. N‐fixation biomarker genes (*nifD*; Supporting Information Table S7) were associated with a filamentous sulfur‐oxidizing genus, *Beggiatoa*, and mesophilic purple sulfur bacterial family, Chromatiaceae.

In comparison, NO reduction was revealed as the most evident biomarker (Figure 2) for UMR sediment microbial communities without mussels, with the abundance of the *norB* gene most responsible for this distinction (Table 2). The *nrfA* gene family was increased without mussels (Table 2), despite both DNRA and NO reduction pathways requiring ${\text{NO}}_{2}^{-}$ as substrate. Although the *nrfA* protein coding gene was characterized as a biomarker of no‐mussel microbial communities, only two *nrfA* genes families (Figure 3d) were increased in abundance. The higher ranking of NO reductase as a no‐mussel biomarker was explained by five differentially increased *norB* genes (Figure 3c), two of which originated from *Ochrobactrum* and *Zoogloea* genera. Numerous other denitrification genes (Supporting Information Table S8) were more abundant without mussels, including 13 different *nosZ* gene families (Figure 3b), six *narG*, and four *nirS* (Figure 3d). A similar number of *narGH* biomarkers were differentially abundant in both treatments while the samples without mussels contained more functional biomarkers for NO reduction, N_2_O reduction, and N‐fixation.

**Table 2:** Biomarker N‐cycling pathways, functional roles, and gene families with no mussels

	N‐cycling function or functional gene	Linear discriminant analysis effect size	*p* value
Level 1: N‐cycle pathway	Denitrification	NA	NA
Level 2: N‐cycle function	Nitric oxide reduction	4.03	0.021
Level 3: functional gene	*norB*	3.88	0.021
Level 1: N‐cycle pathway	DNRA	NA	NA
Level 2: N‐cycle function	Nitrite reduction	NA	NA
Level 3: Functional gene	*nrfA*	3.87	0.043

*Notes*

The abundance of *norB* genes and the higher order functional category of nitric oxide reduction were statistically greater in the no‐mussel treatment, but the denitrification pathway was not significantly different. *nrfA* was statistically more abundant, but the higher order categories of nitrite reduction in the DNRA pathway were not statistically significant.

## 4 DISCUSSION

### 4.1 ${\text{NO}}_{3}^{-}$ and urea metabolism gene families were largely abundant in UMR sediment

Regardless of mussel presence, ${\text{NO}}_{2}^{-}$/${\text{NO}}_{3}^{-}$ redox represented the largest N‐cycling gene family for UMR sediment microbial communities by way of encoding NarGH and NxrAB. This may be explained by reliably high ${\text{NO}}_{3}^{-}$ loads found in the UMR agroecosystem (Kreiling & Houser, 2016), with concentrations measured near 14‐18 mg/L (David et al., 2015; Ikenberry, Soupir, Schilling, Jones, & Seeman, 2014) throughout the UMR watershed. Furthermore, our findings are consistent with previous studies which showed associations between decreased total dissolved N loads in UMR shallow sediments and denitrification rates (Garcia et al., 2016). These results also show consistency with aquatic sediments outside the UMR, where the genomic potential for ${\text{NO}}_{3}^{-}$ reduction and ${\text{NO}}_{2}^{-}$ oxidation dominated oligotrophic sediments while outnumbering the potential for DNRA and anammox in driving N‐cycling (Rasigraf, Schmitt, Jetten, & Luke, 2017).

The large abundance of urea catabolism gene families in both UMR sediment treatments is not surprising. Urea is commonly produced by freshwater fish, microorganisms, human pollution, agricultural runoff, and is typically at highest concentrations near the water–sediment interface (Berman & Bronk, 2003). It is possible that the UMR sediment microbial communities are equipped to degrade urea and ${\text{NO}}_{3}^{-}$ because of high N in the UMR, and the contribution of mussels did not contribute a statistically significant effect on these gene families. Ultimately, this suggests that sediment microbial communities in the UMR have the genetic capability to mitigate urea and ${\text{NO}}_{3}^{-}$, but warrants further research into bioturbation as a technique to enhance the flux of N into sediments and to ultimately reduce non‐point N concentrations.

### 4.2 Nitrification biomarkers in sediments with mussels

**Figure 4:**
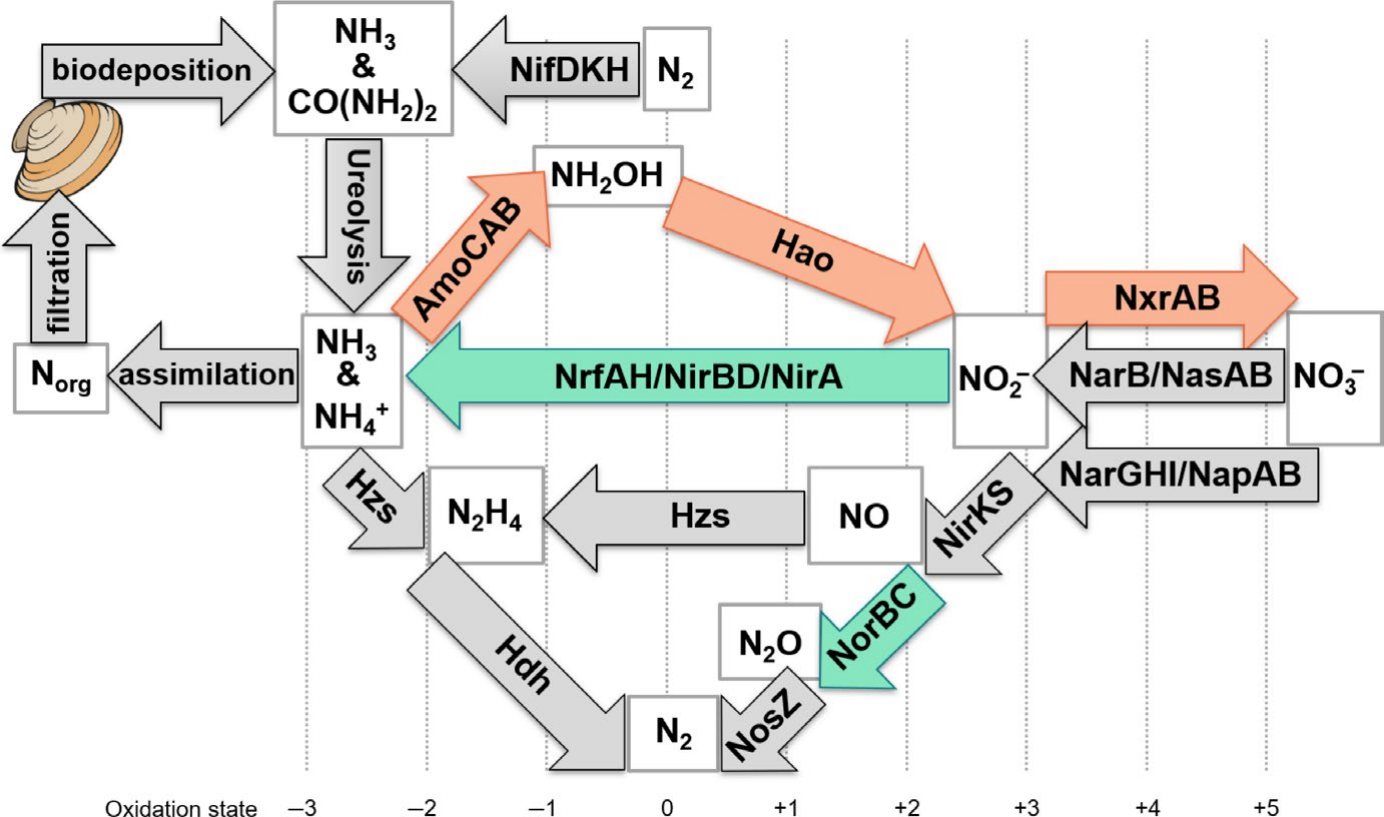
Proposed flow of nitrogen in the Upper Mississippi River freshwater mussel bed. N is added to the sediment by microbial N‐fixation and can be detected by the presence of the functional gene encoding nitrogenase (NifDKH). N may also be added through mussel biodeposition of NH_3_ and urea (CO(NH_2_)_2_), which may be hydrolyzed by urease enzymes. Bioavailable N could be assimilated into microbial biomass or utilized in redox reactions. Assimilated N (N_org_) is recycled in the aquatic system through bivalve filtration processes. Redox transformations of N include microbial nitrification and are quantified by the functional genes encoding, NH_3_ monooxygenase (AmoCAB), hydroxylamine dehydrogenase (Hao), and NO_2_^‐^ oxidoreductase (NxrAB). Complete removal of N is possible with anammox biochemical processes (Hzs, Hdh) or denitrification by sequentially reducing ${\text{NO}}_{3}^{-}$ to N_2_ with reductase enzymes for ${\text{NO}}_{3}^{-}$ (NarGHI/NapAB), ${\text{NO}}_{2}^{-}$ (NirKS), NO (NorBC), and N_2_O (NosZ). Lastly, N may be temporarily sequestered via assimilatory ${\text{NO}}_{3}^{-}$ and ${\text{NO}}_{2}^{-}$ reduction (NasAB/NarB and NirA, respectively) and dissimilatory reduction to NH_3_ (DNRA; NrfAH). Colored arrows represent the biomarker genes found to be differentially abundant in this research without (green) and with mussels (orange)

In confirmation of our hypothesis, the UMR mussel bed sediment contained microbial communities with increased genetic potential for NH_3_ and ${\text{NO}}_{2}^{-}$ oxidation, as well as a greater abundance of the *hao* gene family originating from *Nitrosomonas* (Figure 4). The LEfSe biomarker analysis revealed that nitrification pathways were the most definitive biomarker of N‐cycling microbial communities with mussels and were largely due to increased genetic potential for ${\text{NO}}_{2}^{-}$ oxidation. Furthermore, we identified nitrification biomarkers belonged to the genera *Nitrospira* and *Nitrosomonas* and also matches our previous findings from 16S rRNA amplicon sequencing.

It is possible that mussels had the most impact on nitrification genes because their biodeposition products increase porewater NH_3_ concentrations (Bril et al., 2014) and enhanced the flux of ${\text{NO}}_{3}^{-}$ from water to sediment (Hoellein, Zarnoch, Bruesewitz, & DeMartini, 2017). Other studies have found significantly greater AOB *amoA* genes corresponding with a higher NH_3_ load (Zhang et al., 2016) and aerophilic conditions (Wang et al., 2015). The most distinct nitrification genes were most closely related to NOB *Nitrospira* and comammox *Nitrospira*. It is not surprising that *Nitrospira* species dominated the nitrification biomarkers due to their metabolic diversity (Koch et al., 2015; Lücker et al., 2010), domination within freshwater sediments (Altmann, Stief, Amann, de Beer, & Schramm, 2003), increased abundance in sediments with mussels (Black et al., 2017; Zheng, Tang, Zhang, Qin, & Wang, 2017), and greater abundance from invertebrate bioturbation activities (Shen et al., 2017). Finding *amoA* biomarkers from comammox *Nitrospira* clades suggests that the presence of mussels may enhance the genetic potential for complete nitrification.

### 4.3 Implications of freshwater mussels on N‐cycling

Microbial communities without mussel influences had greater metabolic potential for NO reduction and contained high ranking biomarker genes *norB* and *nrfA* (Figure 4). It makes sense that mussels suppressed the genomic potential for ${\text{NO}}_{2}^{-}$ and NO reduction since these processes are in opposition to ${\text{NO}}_{2}^{-}$ oxidation. Furthermore, our results match a study which found higher abundances of *Nitrospira* near the water–sediment interface of NH_3_‐enriched, mixed and homogenized sediment, at the expense of DNRA (Altmann, Stief, Amann, & de Beer, 2004). The suppression of DNRA by mussels would be an important ecosystem service because DNRA recycles bioavailable N and promotes a positive feedback of eutrophication (Jäntti & Hietanen, 2012). *NrfA* abundance has been positively correlated to sediment C:N concentrations (Lindemann, Zarnoch, Castignetti, & Hoellein, 2016), so it is possible that mussel assemblages lowered this biogeochemical ratio from biodeposition products, and resulted in a suppressed DNRA pathway by microbial communities.

Our main findings of decreased *norB* abundance may be explained by mussel bioturbation activity and aeration of the sediment. One study showed that microaerophilic conditions affect denitrification rates, and decreased *norB* transcripts when O_2_ concentrations exceeded 200 nM (Dalsgaard et al., 2014). Results of decreased genomic potential for NO reduction suggest that mussels could indirectly decrease the production of N_2_O, a potent greenhouse gas (IPCC, 2007, Zhang et al., 2015), in UMR sediments. This is an important finding, as studies have noted that denitrification in the UMR is a major source of atmospheric N_2_O (Turner et al., 2016), and N_2_O emissions from upper Midwest agroecosystem were primarily from soil (Zhang et al., 2015). Turner et al. (2016) also projected that a doubling in aquatic N concentrations would result in a 40% increase in N_2_O emissions from denitrification in the UMR and illustrates that mussels may provide a buffering capacity toward future N_2_O emissions.

## 5 CONCLUSION

Metagenomic sequencing of UMR sediments revealed a large genomic potential for nitrate metabolism and minor abundance of genes for anaerobic NH_3_ oxidation and DNRA pathways. The presence of a well‐established freshwater mussel assemblage in this agroecosystem resulted in significantly increased nitrification potential at the expense of DNRA and NO reduction to N_2_O. In support of these findings, *amoA* and *nxr* genes were the most predominant biomarkers of mussel bed, and the most defining genes were associated with comammox *Nitrospira* and NOB *Nitrospira*, respectively. Additionally, our results provide evidence that mussels may offer a buffer against N_2_O production by suppressing *norB* and prevent a positive feedback for eutrophication via reducing the abundance of *nrfA* genes. Overall, this research demonstrated the genomic potential of N‐cycling microbial communities was impacted by freshwater mussels in a high nutrient agroecosystem.

## CONFLICT OF INTEREST

The authors declare no conflict of interest.

## AUTHOR CONTRIBUTIONS

EMB, MSC, and CLJ contributed to the conception and design of the study; MSC designed the bioinformatic pipeline; EMB performed the statistical analysis; EMB, MSC, and CLJ contributed to the first manuscript draft, manuscript revisions, read and approved the submitted version.

## ETHICS STATEMENT

Care was taken to minimize disturbance to the ecosystem, and no mussels were removed from the study site.

## Data Availability

Our data are publically accessible at the following databases: MG‐RAST project mgp21252 (https://www.mg-rast.org/linkin.cgi?project=mgp21252), the European Nucleotide Archive Study Accession PRJNA414922 (https://www.ebi.ac.uk/ena/data/view/PRJNA414922), and the Sequence Read Archive BioProject ID PRJNA414922 (https://www.ncbi.nlm.nih.gov/sra?linkname=bioproject_sra_all&from_uxml:id=414922).
