# Supplementary figures and images for: Metagenomic analysis of nitrogen‐cycling genes in upper Mississippi river sediment with mussel assemblages
Source: Microbiologyopen. 2018 Oct 1;8(5):e00739. doi: 10.1002/mbo3.739 (PMC6528593; doi:10.1002/mbo3.739)

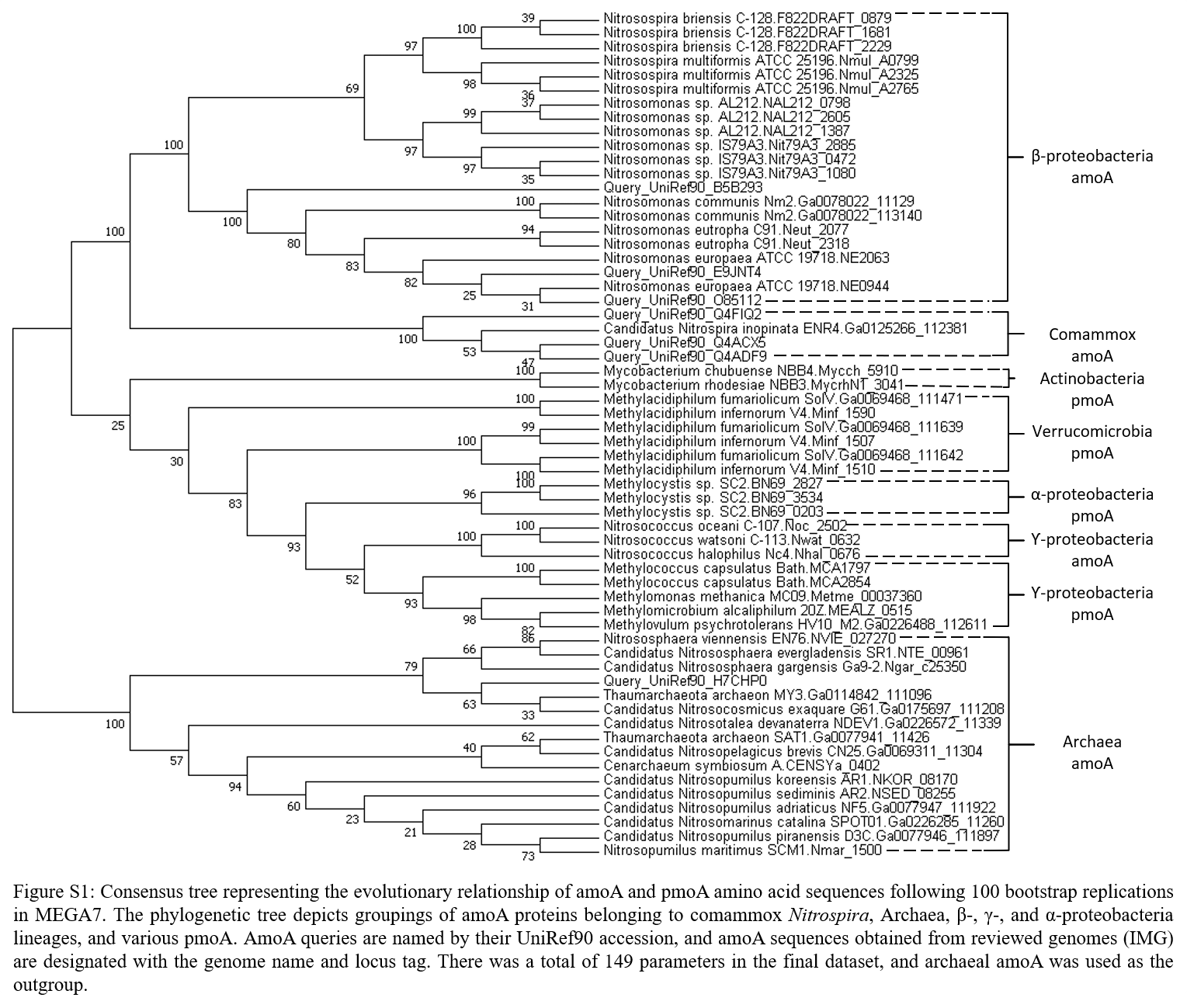

Supplement: Supplementary file 1 [file MBO3-8-e00739-s001.tif]
