# Supplementary material for: Metagenomic analysis of nitrogen‐cycling genes in upper Mississippi river sediment with mussel assemblages
Source: Microbiologyopen. 2018 Oct 1;8(5):e00739. doi: 10.1002/mbo3.739 (PMC6528593; doi:10.1002/mbo3.739)
Supplement: Supplementary file 2 [file MBO3-8-e00739-s002.docx]

Supplementary Tables

Metagenomic analysis of nitrogen cycling genes in upper Mississippi river sediment with mussel assemblages

Ellen M. Black, Michael S. Chimenti, Craig L. Just

Table S1: Sequence accessions for each sample found at MG-RAST, ENA, and NCBI. MG-RAST projects detail sequence statistics from QA/QC, and link this shotgun sequencing study with previous amplicon sequencing results.

| Sample Name | MG-RAST ID | MG-RAST  (16S rRNA amplicon) | European Nucleotide Archive and NCBI Sequence Read Archive Accession |
| --- | --- | --- | --- |
|  | MG-RAST Project mgp21252 | MG-RAST Project mgp18682 | Study Accession/BioProject ID PRJNA414922 |
| No mussel (S1) | mgm4730047.3 | mgm4705698.3 | SAMN06710719 |
| No mussel (S2) | mgm4730043.3 | mgm4705704.3 | SAMN06710724 |
| No mussel (S3) | mgm4730044.3 | mgm4705686.3 | SAMN06710731 |
| No mussel (S4) | mgm4730042.3 | mgm4705697.3 | SAMN06710729 |
| Mussel (S5) | mgm4730045.3 | mgm4705708.3 | SAMN06710710 |
| Mussel (S6) | mgm4730048.3 | mgm4705672.3 | SAMN06710716 |
| Mussel (S7) | mgm4730041.3 | mgm4705699.3 | SAMN06710705 |
| Mussel (S8) | mgm4730046.3 | mgm4705680.3 | SAMN06710725 |

Table S2: Protein clusters that mapped onto KEGG orthologous groups within the Nitrogen metabolism module and urease functions were considered for this study. Parent categories only contained functional genes unique to the given module to obtain discrete categories. As a result, NarGHI and NapAB were specific to dissimilatory nitrate reduction and were not included in denitrification or DNRA pathways. The full functional “tree” used for LDA can be found at https://metacyc.org/group?id=biocyc13-27028-3726046429-frozen.

The complete list of KO and GO relative abundance profiles for our metagenomes can be found at the following links: https://metacyc.org/group?id=biocyc13-27028-3725982783 and https://metacyc.org/group?id=biocyc13-27028-3725983191, respectively.

| KEGG Nitrogen metabolism modules | KO and protein name | | | |
| --- | --- | --- | --- | --- |
| nitrogen fixation | nitrogenase | | nitrogenase delta subunit | |
|  | NifDKH | | AnfG | |
|  | K002586, K002588, K002591 | | K00531 | |
| assimilatory nitrate reduction | ferredoxin-nitrate reductase | | ferredoxin-nitrite reductase | |
|  | NarB | | NirA | |
|  | K00367 | | K00366 | |
| dissimilatory nitrate reduction | nitrate reductase | | periplasmic nitrate reductase | |
|  | NarGHI | | NapAB | |
|  | K00370, K00371, K00374 | | K02567-K02568 | |
| denitrification | nitrite reductase (NO-forming) | nitrite reductase (NO-forming)/hydroxylamine reductase | nitric oxide reductase | nitrous-oxide reductase |
|  | NirK | NirS | NorBC | NosZ |
|  | K00368 | K15864 | K04561 | K00376 |
| nitrification | nitrite oxidoreductase | hydroxylamine oxidoreductase | ammonia monooxygenase | |
|  | NxrAB | Hao | AmoCAB | |
|  | K00370, K00371, K00374 | K10535 | K10944-K10946 | |
| anammox | nitrite reductase (NO-forming) | nitrite reductase (NO-forming)/  hydroxylamine reductase | hydrazine hydrolase | hydrazine synthase |
|  | NirK | NirS | Hdh | Hzs |
|  | K00368 | K15864 | K20935 | K20932-K20934 |
| urease | urease subunit alpha | urease subunit beta | urease subunit gamma | |
|  | UreC | UreB | UreA | |
|  | K01428 | K01429 | K01430 | |
| DNRA | nitrite reductase (NADH) | | nrfA; nitrite reductase (cytochrome c-552) | |
|  | NirBD | | NrfAH | |
|  | K00362-K00363 | | K03385 | |

Table S3: Differentially abundant nitrification and urease protein clusters listed in order of decreasing effect size. All nitrification and urease genes deemed differentially abundant were greater in the mussel treatment.

| N-cycling functional gene | Protein cluster (LCA) | LDA effect size | P value | Treatment |
| --- | --- | --- | --- | --- |
| *nxrC* | UniRef90_D8PI74 (*Nitrospira*) | 3.32 | 0.018 | Mussel |
| *nxrB* | UniRef90_D8PI40 (*Nitrospira*) | 3.21 | 0.021 | Mussel |
| *nxrA* | UniRef90_D8PI59 (*Nitrospira*) | 3.16 | 0.021 | Mussel |
| *amoA* | UniRef90_Q4FIQ2 (Bacteria) | 3.07 | 0.043 | Mussel |
| *amoA* | UniRef90_Q4ACX5 (Bacteria) | 2.96 | 0.043 | Mussel |
| *nxrB* | UniRef90_T2HGM4 (Bacteria) | 2.96 | 0.043 | Mussel |
| *ureC* | UniRef90_D2CR64 (unclassified) | 2.96 | 0.021 | Mussel |
| *amoA* | UniRef90_Q4ADF9 (Bacteria) | 2.79 | 0.018 | Mussel |
| *nxrA* | UniRef90_D8PI41 (*Nitrospira*) | 2.75 | 0.021 | Mussel |
| *hao* | UniRef90_B9V947 (*Nitrosomonas*) | 2.46 | 0.043 | Mussel |
| *ureC* | UniRef90_D2CR19 (unclassified) | 2.43 | 0.043 | Mussel |
| *ureC* | UniRef90_D2CQ18 (unclassified) | 2.41 | 0.038 | Mussel |
| *amoA* | UniRef90_Q04507 (Bacteria) | 2.39 | 0.047 | Mussel |
| *amoA* | UniRef90_O85112 (Bacteria) | 2.37 | 0.047 | Mussel |
| *amoA* | UniRef90_B5B293 (Bacteria) | 2.33 | 0.047 | Mussel |
| *amoA* | UniRef90_H7CHP0 (Archaea) | 2.32 | 0.047 | Mussel |
| *amoA* | UniRef90_E9JNT4 (Bacteria) | 2.26 | 0.047 | Mussel |

Table S4: List of IMG bacterial and archaeal completed genomes and respective monooxygenase gene products used for multiple sequence alignments.

| **Genome Name** | **Genome ID** | **Gene Product** | **Gene ID** | **Locus Tag** |
| --- | --- | --- | --- | --- |
| *Candidatus* Nitrosocosmicus exaquare G61 | 2718217702 | amoA | 2718536227 | Ga0175697_111208 |
| *Candidatus* Nitrosomarinus catalina SPOT01 | 2757320762 | amoA | 2758441363 | Ga0226285_11260 |
| *Candidatus* Nitrosopelagicus brevis CN25 | 2630968793 | amoA | 2633271616 | Ga0069311_11304 |
| *Candidatus* Nitrosopumilus adriaticus NF5 | 2627854092 | amoA | 2630190648 | Ga0077947_111922 |
| *Candidatus* Nitrosopumilus koreensis AR1 | 2518645532 | amoA | 2518760421 | NKOR_08170 |
| *Candidatus* Nitrosopumilus piranensis D3C | 2627853696 | amoA | 2628584258 | Ga0077946_111587 |
| *Candidatus* Nitrosopumilus piranensis D3C | 2627853696 | amoA | 2628584568 | Ga0077946_111897 |
| *Candidatus* Nitrosopumilus sediminis AR2 | 2518645576 | amoA | 2518891990 | NSED_08255 |
| *Candidatus* Nitrososphaera evergladensis SR1 | 2585427666 | amoA | 2586120464 | NTE_00961 |
| *Candidatus* Nitrososphaera gargensis Ga9-2 | 2510065023 | amoA | 2510154470 | Ngar_c25350 |
| *Candidatus* Nitrosotalea devanaterra NDEV1 | 2757320681 | amoA | 2758412811 | Ga0226572_11339 |
| *Cenarchaeum symbiosum A* | 641522613 | amoA | 641734427 | CENSYa_0402 |
| *Nitrosopumilus maritimus SCM1* | 641228499 | amoA | 641317151 | Nmar_1500 |
| *Nitrososphaera viennensis EN76* | 2585427612 | amoA | 2585919001 | NVIE_027270 |
| *Thaumarchaeota archaeon MY3* | 2657244923 | amoA | 2657390414 | Ga0114842_111096 |
| *Thaumarchaeota archaeon SAT1* | 2630968650 | amoA | 2632674335 | Ga0077941_11426 |
| *Nitrosomonas communis Nm2* | 2627854142 | amoA | 2630388647 | Ga0078022_11129 |
| *Nitrosomonas communis Nm2* | 2627854142 | amoA | 2630391654 | Ga0078022_113140 |
| *Nitrosomonas europaea ATCC 19718* | 637000195 | amoA | 637427314 | NE0944 |
| *Nitrosomonas europaea ATCC 19718* | 637000195 | amoA | 637428388 | NE2063 |
| *Nitrosomonas eutropha C91* | 637000196 | amoA | 638132300 | Neut_2077 |
| *Nitrosomonas eutropha C91* | 637000196 | amoA | 638132536 | Neut_2318 |
| *Nitrosomonas sp. AL212* | 650716066 | amoA | 650753375 | NAL212_0798 |
| *Nitrosomonas sp. AL212* | 650716066 | amoA | 650753934 | NAL212_1387 |
| *Nitrosomonas sp. AL212* | 650716066 | amoA | 650755078 | NAL212_2605 |
| *Nitrosomonas sp. IS79A3* | 650716067 | amoA | 651001907 | Nit79A3_0472 |
| *Nitrosomonas sp. IS79A3* | 650716067 | amoA | 651002509 | Nit79A3_1080 |
| *Nitrosomonas sp. IS79A3* | 650716067 | amoA | 651004224 | Nit79A3_2885 |
| *Nitrosospira briensis C-128* | 2585428181 | amoA | 2588205525 | F822DRAFT_0879 |
| *Nitrosospira briensis C-128* | 2585428181 | amoA | 2588206327 | F822DRAFT_1681 |
| *Nitrosospira briensis C-128* | 2585428181 | amoA | 2588206875 | F822DRAFT_2229 |
| *Nitrosospira multiformis ATCC 25196* | 637000197 | amoA | 637810885 | Nmul_A0799 |
| *Nitrosospira multiformis ATCC 25196* | 637000197 | amoA | 637812413 | Nmul_A2325 |
| *Nitrosospira multiformis ATCC 25196* | 637000197 | amoA | 637812857 | Nmul_A2765 |
| *Candidatus Nitrospira inopinata ENR4* | 2684623072 | amoA | 2686679860 | Ga0125266_112381 |
| *Nitrosococcus halophilus Nc4* | 646564556 | amoA | 646691322 | Nhal_0676 |
| *Nitrosococcus oceani C-107* | 637000194 | amoA | 637738981 | Noc_2502 |
| *Nitrosococcus watsoni C-113* | 648028046 | amoA | 648071294 | Nwat_0632 |
| *Mycobacterium chubuense NBB4* | 2506783014 | pmoA | 2506875039 | Mycch_5910 |
| *Mycobacterium rhodesiae NBB3* | 2508501106 | pmoA | 2509042925 | MycrhN1_3041 |
| *Methylocystis sp. SC2* | 2540341127 | pmoA | 2540711774 | BN69_0203 |
| *Methylocystis sp. SC2* | 2540341127 | pmoA | 2540714425 | BN69_2827 |
| *Methylocystis sp. SC2* | 2540341127 | pmoA | 2540715141 | BN69_3534 |
| *Methylococcus capsulatus Bath* | 637000166 | pmoA | 637170957 | MCA1797 |
| *Methylococcus capsulatus Bath* | 637000166 | pmoA | 637171983 | MCA2854 |
| *Methylomicrobium alcaliphilum 20Z* | 2540341096 | pmoA | 2540614434 | MEALZ_0515 |
| *Methylomonas methanica MC09* | 2504756059 | pmoA | 2504962877 | Metme_00037360 |
| *Methylovulum psychrotolerans HV10_M2* | 2757320928 | pmoA | 2758521633 | Ga0226488_112611 |
| *Methylacidiphilum fumariolicum SolV* | 2630968640 | pmoA | 2632639552 | Ga0069468_111471 |
| *Methylacidiphilum fumariolicum SolV* | 2630968640 | pmoA | 2632639720 | Ga0069468_111639 |
| *Methylacidiphilum fumariolicum SolV* | 2630968640 | pmoA | 2632639723 | Ga0069468_111642 |
| *Methylacidiphilum infernorum V4* | 642555138 | pmoA | 642665737 | Minf_1507 |
| *Methylacidiphilum infernorum V4* | 642555138 | pmoA | 642665740 | Minf_1510 |
| *Methylacidiphilum infernorum V4* | 642555138 | pmoA | 642665821 | Minf_1590 |

Table S5: Biomarker gene families composing the dissimilatory nitrate reduction pathway. Specific protein clusters are listed in decreasing order of LDA effect size and are labeled with the treatment containing the greater abundance. The higher order pathway, denitrification, was not deemed differentially abundant.

| N-cycling functional gene | Protein cluster (LCA) | LDA effect size | P value | Treatment |
| --- | --- | --- | --- | --- |
| *narH* | UniRef90_UPI00035DFE69 (*Methylosarcina*) | 2.81 | 0.018 | Mussel |
| *narG* | UniRef90_A9Y2C8 (Bacteria) | 2.80 | 0.047 | No mussel |
| *narG* | UniRef90_D5KJU2 (Bacteria) | 2.78 | 0.047 | No mussel |
| *narG* | UniRef90_B6E5A4 (Bacteria) | 2.65 | 0.042 | No mussel |
| *narH* | UniRef90_UPI0004057A01 (*Pleomorphomonas*) | 2.57 | 0.018 | No mussel |
| *narG* | UniRef90_B6E5P2 (Bacteria) | 2.53 | 0.043 | Mussel |
| *narG* | UniRef90_G3J0V4 (*Methylobacter*) | 2.38 | 0.043 | Mussel |
| *narG* | UniRef90_C0M0N4 (Bacteria) | 2.21 | 0.021 | No mussel |
| *narG* | UniRef90_H0TSP6 (*Bradyrhizobium*) | 2.20 | 0.021 | No mussel |
| *narG* | UniRef90_B1G0C2 (*Burkholderia*) | 2.19 | 0.043 | Mussel |
| *narG* | UniRef90_B1PUT8 (Bacteria) | 2.19 | 0.043 | Mussel |

Table S6: Differentially abundant protein clusters within the parent category, DNRA. These differentially abundant genes are listed in order of decreasing effect size and the treatment containing the greater abundance.

| N-cycling functional gene | Protein cluster (LCA) | LDA effect size | P value | Treatment |
| --- | --- | --- | --- | --- |
| *nrfA* | UniRef90_S4UCW4 (Bacteria) | 3.22 | 0.014 | No mussel |
| *nrfA* | UniRef90_S4UD05 (Bacteria) | 2.79 | 0.047 | No mussel |
| *nirB* | UniRef90_V5BZ33 (Methyloglobulus) | 2.94 | 0.014 | Mussel |
| *nrfA* | UniRef90_S4UG91 (Bacteria) | 2.46 | 0.047 | Mussel |

Table S7: N-fixation functional genes were differentially abundant within both treatments. The genes are listed in order of decreasing effect size and correspond to the treatment with greater abundance.

| N-cycling functional gene | Protein cluster (LCA) | LDA effect size | P value | Treatment |
| --- | --- | --- | --- | --- |
| *nifD* | UniRef90_B4ULZ0 (*Anaeromyxobacter*) | 2.65 | 0.043 | No mussel |
| *nifD* | UniRef90_A7HET0 (*Anaeromyxobacter*) | 2.64 | 0.043 | No mussel |
| *nifH* | UniRef90_Q4PS30 (unclassified) | 2.53 | 0.047 | No mussel |
| *nifH* | UniRef90_E1QE30 (*Desulfarculus*) | 2.43 | 0.020 | No mussel |
| *nifH* | UniRef90_W5U1A9 (unclassified) | 2.42 | 0.047 | Mussel |
| *nifH* | UniRef90_G8B3A2 (Bacteria) | 2.41 | 0.018 | No mussel |
| *nifH* | UniRef90_B7ZGG9 (Alphaproteobacteria) | 2.36 | 0.014 | No mussel |
| *nifH* | UniRef90_Q8KKJ6 (Bacteria) | 2.36 | 0.042 | Mussel |
| *nifH* | UniRef90_B6DAC8 (Bacteria) | 2.33 | 0.020 | No mussel |
| *nifD* | UniRef90_D3RUE5 (Chromatiaceae) | 2.30 | 0.047 | Mussel |
| *nifH* | UniRef90_X2IXN1 (Bacteria) | 2.30 | 0.047 | Mussel |
| *nifH* | UniRef90_D3H5I8 (Bacteria) | 2.28 | 0.047 | No mussel |
| *nifH* | UniRef90_X2J0I1 (Bacteria) | 2.28 | 0.047 | No mussel |
| *nifD* | UniRef90_I3CFW7 (*Beggiatoa*) | 2.24 | 0.047 | Mussel |

Table S8: Denitrification gene families were differentially abundant despite the denitrification pathway not being statistically different. Treatment label indicates if mussels or no-mussels were increased in abundance.

| N-cycling functional gene | Protein cluster (LCA) | LDA effect size | P value | Treatment |
| --- | --- | --- | --- | --- |
| **Nitrous oxide reduction** |  |  |  |  |
| *nosZ* | UniRef90_K7XSE7 (Bacteria) | 2.76 | 0.021 | No mussel |
| *nosZ* | UniRef90_K7Y664 (Bacteria) | 2.70 | 0.047 | No mussel |
| *nosZ* | UniRef90_K7XTV0 (Bacteria) | 2.68 | 0.047 | No mussel |
| *nosZ* | UniRef90_K7XMD0 (Bacteria) | 2.65 | 0.020 | No mussel |
| *nosZ* | UniRef90_K7WLZ2 (unclassified) | 2.65 | 0.047 | No mussel |
| *nosZ* | UniRef90_B8R0E4 (Bacteria) | 2.59 | 0.047 | No mussel |
| *nosZ* | UniRef90_K7XU27 (Bacteria) | 2.57 | 0.020 | No mussel |
| *nosZ* | UniRef90_K7X6F4 (Bacteria) | 2.57 | 0.047 | No mussel |
| *nosZ* | UniRef90_B8R0A6 (Bacteria) | 2.48 | 0.043 | Mussel |
| *nosZ* | UniRef90_K7X032 (Bacteria) | 2.45 | 0.038 | No mussel |
| *nosZ* | UniRef90_A1KA74 (Azoarcus) | 2.43 | 0.047 | No mussel |
| *nosZ* | UniRef90_K7XCX6 (unclassified) | 2.40 | 0.042 | No mussel |
| *nosZ* | UniRef90_B8R0B8 (Bacteria) | 2.37 | 0.043 | Mussel |
| *nosZ* | UniRef90_S5UJ63 (Bacteria) | 2.27 | 0.020 | Mussel |
| *nosZ* | UniRef90_H9BVI3 (Bacteria) | 2.24 | 0.047 | No mussel |
| **Nitric oxide reduction** |  |  |  |  |
| *norB* | UniRef90_F8S9B7 (Bacteria) | 2.94 | 0.047 | No mussel |
| *norB* | UniRef90_F8S960 (Bacteria) | 2.87 | 0.038 | No mussel |
| *norB* | UniRef90_F8S8X5 (Bacteria) | 2.73 | 0.047 | No mussel |
| *norB* | UniRef90_G2FJZ6 (Gammaproteobacteria) | 2.49 | 0.047 | Mussel |
| *norB* | UniRef90_I1ZIV3 (*Zoogloea*) | 2.46 | 0.021 | No mussel |
| *norB* | UniRef90_C5NT92 (*Ochrobactrum*) | 2.39 | 0.047 | No mussel |
| *norB* | UniRef90_V9TN78 (Proteobacteria) | 2.36 | 0.047 | Mussel |
| *norB* | UniRef90_L0PRW7 (unclassified) | 2.35 | 0.047 | Mussel |
| *norB* | UniRef90_Q84D93 (Bacteria) | 2.21 | 0.042 | Mussel |
| **Nitrite reduction** |  |  |  |  |
| *nirS* | UniRef90_B2CG04 (Bacteria) | 2.54 | 0.043 | No mussel |
| *nirS* | UniRef90_A6YLH2 (Bacteria) | 2.53 | 0.047 | No mussel |
| *nirS* | UniRef90_Q59HK1 (Bacteria) | 2.47 | 0.047 | Mussel |
| *nirS* | UniRef90_G0Z4D9 (Bacteria) | 2.44 | 0.021 | No mussel |
| *nirS* | UniRef90_F6IBN8 (Bacteria) | 2.41 | 0.047 | Mussel |
| *nirS* | UniRef90_A0A024BRU3 (Bacteria) | 2.38 | 0.047 | Mussel |
| *nirS* | UniRef90_Q2F353 (Bacteria) | 2.37 | 0.047 | Mussel |
| *nirS* | UniRef90_A0A024BR71 (Bacteria) | 2.30 | 0.047 | Mussel |
| *nirS* | UniRef90_Q6TBA1 (Bacteria) | 2.22 | 0.020 | No mussel |
| *nirS* | UniRef90_A0A024BS11 (Bacteria) | 2.22 | 0.021 | Mussel |
| *nirS* | UniRef90_G4XN08 (Bacteria) | 2.19 | 0.042 | Mussel |
